# Retention of the Aboriginal Health, Ageing, and Disability Workforce: Protocol for a Mixed Methods Study

**DOI:** 10.2196/25261

**Published:** 2021-05-28

**Authors:** John Gilroy, Kim Bulkeley, Folau Talbot, Josephine Gwynn, Kylie Gwynne, Mandy Henningham, Caroline Alcorso, Boe Rambaldini, Michelle Lincoln

**Affiliations:** 1 The University of Sydney Sydney, NSW Australia

**Keywords:** Indigenous health, disability, ageing, Indigenous methodologies, Indigenous, Australia, Aboriginal

## Abstract

**Background:**

Despite a plethora of research into Aboriginal employment and recruitment, the extent and nature of the retention of frontline Aboriginal people in health, ageing, and disability workforces are currently unknown. In this application, frontline service delivery is defined as Aboriginal people who are paid employees in the health, ageing, and disability service sectors in roles that involve direct client, participant, or patient contact. There is a need to identify the factors that inhibit (push) and promote (pull) staff retention or departure of this workforce from the sectors. This study will provide additional insight about this topic.

**Objective:**

The objective of this project is to uncover the factors that influence the retention of frontline Aboriginal workers in the health, ageing, and disability workforces in New South Wales (NSW) who do not have university qualifications. The aim of the proposed project aims to discover the push and pull factors for the retention of the frontline Aboriginal workforce in the health, ageing, and disability sectors in NSW in relation to their role, employment, and community and design evidence-based strategies for retaining the Aboriginal frontline workforce in the health, ageing, and disability sectors in NSW.

**Methods:**

The proposed research will use a mixed methods approach, collecting both quantitative and qualitative data via surveys and interviews to capture and represent the voices and perspectives of Aboriginal people in a way that the participants chose.

**Results:**

Indigenous research methodologies are a growing field in Aboriginal health research in Australia. A key strength of this study is that it is led by Aboriginal scholars and Aboriginal controlled organizations that apply an Indigenous methodological framework throughout the research process.

**Conclusions:**

This study uses a mixed methods design. The survey and interview questions and model were developed in partnership with Aboriginal health, ageing, and disability service workers rather than relying only on research publications on the workforce, government policies, and human resources strategies. This design places a strong emphasis on generalizable findings together with an inductive approach that explores employers and workers’ lived experience of the Aboriginal health workforce in NSW. Excluding workers who have graduated from university places a strong focus on the workforce who have obtained either school or Technical and Further Education or registered training organizations qualifications. Data collection was conducted during the COVID-19 pandemic, and results will include the unique experiences of Aboriginal workers and employers delivering services in an extremely challenging organizational, community, and personal context.

**International Registered Report Identifier (IRRID):**

PRR1-10.2196/25261

## Introduction

### Background

Despite Australia being one of the most developed nations in the world, there is a significant and widening gap in health and welfare between Aboriginal and Torres Strait Islander (Aboriginal) people and non-Aboriginal people. Aboriginal people experience significantly higher rates of chronic health conditions, preventable disease, and disability than non-Aboriginal people because of the effects of colonization and structural barriers to accessing health services. The poor rates of health and well-being are an identified cause of Aboriginal life expectancy being around 10 years lower than that of non-Aboriginal people [1].

During the Aboriginal rights movement, Aboriginal communities built the Aboriginal workforce in health and social services to capitalize on Aboriginal cultures and knowledge to close the gap in life expectancy, health, and well-being between Aboriginal and non-Aboriginal people. An Aboriginal workforce is essential for community self-determination, community governance, and the design and delivery of culturally safe services. A vibrant Aboriginal community-controlled health sector including a substantial Aboriginal workforce has a significant positive influence on Aboriginal families’ health and well-being [2,3].

Often, Aboriginal workers known or local to Aboriginal communities who access the health, ageing, and disability supports play a vital role in guiding their non-Aboriginal colleagues in ways to adapt their interactions, advice, and interventions to ensure that they are culturally appropriate and safe for Aboriginal patients and clients. These additional responsibilities that Aboriginal workers in the welfare, health, disability, and aged care systems hold as cultural interpreters and practitioners are typically not included in position descriptions or recognized as an essential part of their role [4,5]. This can create issues in the workplace with respect to roles and challenges for Aboriginal staff trying to have the services sector recognize their cultural obligations as part of their personal and professional roles in the local community. The Aboriginal health workforce can increase access and engagement with service providers and facilitate better health, education, and quality of life outcomes for Aboriginal people [6]. This is supported by leading groups who suggest that an Aboriginal workforce is effective in promoting access and engagement with health interventions and providing support for people with disabilities through the provision of culturally safe services and practices [7,8].

Both national and international peak health and disability organizations have called for further development and improved retention of Aboriginal workforces to address the existing social, health, and well-being disparities experienced by Aboriginal people worldwide [9,10]. A recent government report on the Aboriginal health workforce stated that “improved opportunities for employment, advancement, and retention also require attention” [11]. It is not sufficient to simply increase the number of Aboriginal staff. Explicit strategies need to be in place so that workplaces are culturally safe and the roles Aboriginal people play as cultural brokers are recognized and valued.

Many scholars have previously highlighted the shortcomings of the current data available on the retention of the health workforce [12-14]. Although there is growing literature, including systematic literature reviews [13,15-17], on the recruitment and retention of the general rural and remote health workforce, there is very minimal research on Aboriginal frontline service at a national level or at state or territory levels [13,16]. Russell et al [14] found that Aboriginal health practitioners working in remote areas of the Northern Territory had a significantly high turnover rate, concluding that the mean annual turnover rates for nurses and Aboriginal health practitioners combined were extremely high, irrespective of whether turnover was defined as no longer working in any remote clinic (66%) or no longer working at a specific remote clinic (128%). Stability rates were low, and only 20% of nurses and Aboriginal health practitioners remained working at a specific remote clinic 12 months after commencing. Half of them left within 4 months.

The report by Russell et al [14] on rural and remote health service providers found that the annual turnover rate for Aboriginal health workers was 20.8% per year compared with 11.5% for doctors and 13.8% for nurses. Russell et al [14] concluded that their evidence suggests that benchmarks for median survival for the different disciplines in rural and remote areas over a 12-month period in 2008 were as given in Table 1.

**Table 1:** Staff turnover indicator by remoteness and discipline in 2008.

Professional role names	Rural (years)	Remote (years)
Rural remote nurses	5	3.5
Doctors	3	2
Allied health professionals	3	2
Aboriginal health workers	3	3
Managers	5	3.5

A recent national survey of small rural and remote health services by Humphreys et al [12] concluded that the costs for recruiting Aboriginal health workers ranged from Aus $3534 (US $ 2728.87) to Aus $43,600 (US $ 33666.83) during 2008.

Recent systematic literature reviews of Aboriginal health and welfare workforce research found that the experiences of Aboriginal people in the health workforce were affected by their engagement with culturally incompetent staff and managers, education, training, and employment. These factors also affect the success and longevity of the non-Aboriginal workforce working in Aboriginal health; for example, attitudes and behaviors of the workforce have a direct effect on service delivery and design. These studies [13,15,16] suggested many strategies to improve retention of Aboriginal workers, such as ongoing training and support for non-Aboriginal health workers, culturally appropriate service design, and effective tertiary student placements.

Similar conclusions were shared by Rose and Jackson Pulver [17] in their earlier review of existing literature on parallel work by Aboriginal health workers and the health promotion needs of Aboriginal communities. The existing vocational training provision for Aboriginal health workers and the potential role of university-based programs to further Aboriginal health worker professional qualifications to a level equivalent to allied health professionals was explored. This study advocated that Aboriginal health workers require sophisticated skills and knowledge at a level equivalent to other health professions to succeed in this area with a recommendation that opportunities should be offered to Aboriginal health workers, including opportunities at a university level in parallel with other health professional qualifications [17].

When investigating the extent and nature of the retention of Aboriginal frontline workers, it is important to look at external factors such as education and career development. Tertiary education providers, such as universities and Technical and Further Education (TAFE) institutes, and registered training organizations have policies and initiatives that recruit and support the education of Aboriginal students. However, despite this focus at a policy and practical level across sectors, the Aboriginal health and disability workforce is growing at a very slow rate and will not meet the needs of the future. Currently, there are more employment opportunities in the health and disability sectors than there are Aboriginal people who are appropriately qualified to fill them [18]. In 2013, New South Wales (NSW) TAFE reported that the completion rates for Aboriginal students across all qualifications were less than 30% [19].

A scholarship program for Aboriginal preregistration nursing students used five enablers for success [20] to attempt to address completion rates. This study, using structured interviews with students and staff, examined whether these enablers were associated with academic success. A total of 64.5% of the students (n=20) and 75% of the staff (n=6) participated in the study, and it was found that the five enablers were contributing factors to success. This included individual student characteristics; academics’ knowledge, understanding, and awareness; connections, partnerships, and relationships; institutional systems, structures, and processes; and finally, family and community knowledge, understanding, and awareness [20]. Gwynne et al [19] adapted this model for vocational education and identified 2 more enablers: (1) employer support and (2) listening and improving. This program achieved a completion rate of 96.8% using the seven enablers. To further improve completion rates for Aboriginal students, vocational education programs need to be customized to cultural, family, and community contexts. This highlights two points of interest: experiences of success for future Aboriginal health care students and that the environments that customize the student (or worker) experience to a cultural, family, and community context have beneficial results. Ongoing engagement in training may be a key factor in the retention of Aboriginal health care *students* in Aboriginal frontline *health care roles*.

### Objectives

There is a need to identify the factors that inhibit (push) and promote (pull) staff retention or departure of this workforce from various sectors. This study aims to identify the barriers and enablers to retaining Aboriginal people who do not have university qualifications in the health, disability, and aged care workforces. In this paper, *frontline service delivery* is defined as Aboriginal staff who are paid employees in the health, ageing, disability, and community service sectors in roles that involve direct client, participant, or patient support.

For further context, this paper adopts the definition of retention as given by Humphreys et al [12] as the length of time between commencement and termination of employment and turnover as the number of terminations in a specified time period.

Historically, research involving Indigenous people around the world was situated on ethnocentric and Eurocentric values and ideals of Indigenous people that served the interests of the Western imperial elite classes [21,22]. This study will apply an Indigenous research methodological framework [23]. Indigenous research methodologies aim to deconstruct the Western research paradigm by prioritizing local Indigenous community social and cultural values of research and knowledge production [23,24]. In the context of this framework, we briefly describe the standpoint of each author of this paper. The authors are all team members on the project, which includes Aboriginal and non-Aboriginal researchers, 3 Aboriginal community-controlled organizations, and 1 disability service peak body. Four of the authors, JG, BR, FT, and MH, are Aboriginal academics with experience in health workforce research. The other authors are non-Indigenous scholars who have long-standing engagement in Aboriginal health workforce research.

## Methods

### Overview

Through the global Indigenous rights movement, Indigenous advocates have obtained greater control over the research agenda, placing them in a unique position to investigate and decolonize the research processes. This transformation has resulted in a change from a *researcher-directed* approach to *Indigenous community–directed* and *Indigenous persons’ research–directed* approach [25,26]. In effect, the responsibility and accountability for Indigenous research has shifted from North-metropole academic institutions to Indigenous scholars, Indigenous community-controlled organizations, and Indigenous representative bodies.

This study is a mixed methods design involving an Indigenous decolonizing methodological framework that drives all phases of the research. Decolonization centers on privileging the needs of Aboriginal people by analyzing and dismantling the power imbalances that exist between Indigenous people and non-Indigenous people in how research is undertaken to inform government policy, practice, and praxis. This study reflects the decolonizing models developed by Aboriginal scholars Rigney [27] and Gilroy [22,28] for Indigenous research.

1. The research is counterhegemonic to Western ideologies and promotes Indigenous people’s self-determination in the production of knowledge.
2. The research privileges Indigenous voices.
3. The research has Indigenous people involved in the research as researchers.

Our model is influenced by other approaches to the conduct of research on Aboriginal communities [29,30]. These approaches privilege Aboriginal community leadership and governance in all phases of research, including development, implementation, data analysis, and interpretation of results [30]. Community governance is ensured through the structures of advisory and reference groups, processes and procedures of the research, and formalized agreements where required. These approaches vary among communities to reflect the diversity of Aboriginal communities.

### Ethics and Governance

**Figure 1:**
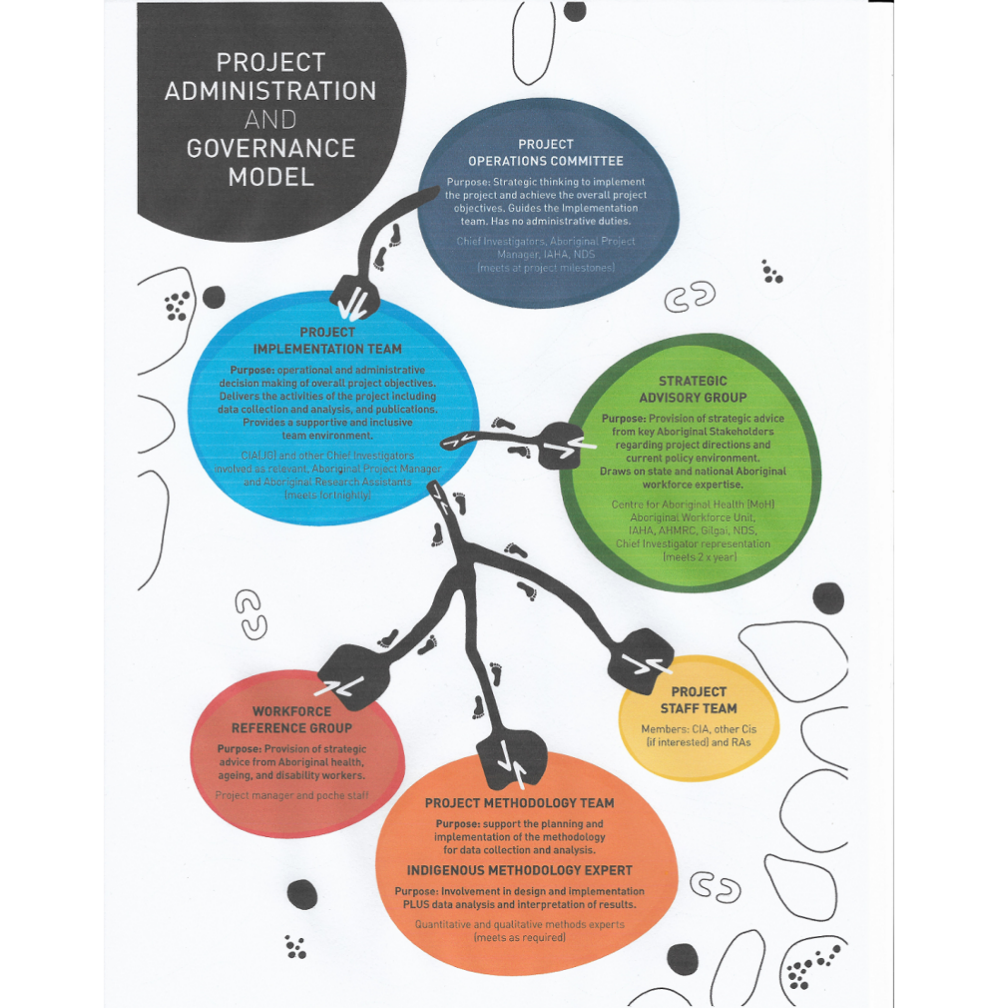
Project administration and governance model. AHMRC: Aboriginal Health and Medical Research Council. CIA: chief investigator A. IAHA: Indigenous Allied Health Australia. NDS: National Disability Services. RA: research assistant.

Ethical approval was received from the Aboriginal Health and Medical Research Council of NSW (1505/19) and the NSW Ministry of Health ethics committee (2019/ETH08775). The study applies the National Health and Medical Research Council (NHMRC) guidelines and principles for Aboriginal research [29,31]. Reflecting the principles of Indigenous research methodologies, the project will be managed using an Aboriginal governance model, as depicted in Figure 1. This model is informed by previous Aboriginal community-led research that involved this project’s researchers [31]. However, this representation is not hierarchical and more faithfully aligns with an Indigenous decolonizing methodological approach with a yarning circle at the center and smaller yarning circles surrounding this, involving the various groups and committees who contribute to decision making and idea formation.

The project implementation team primarily consists of Aboriginal research project manager (FT) and the chief investigator A (JG), whose role is to coordinate the project, and an Aboriginal person recruited as a part-time research assistant. This model privileges Indigenous voices in the process of project planning and implementation. The operations committee, consisting of agency partners and researchers on the project, will guide the thinking and delivery of the project. The implementation team consists of project researchers, including the project manager and research assistants, whose roles are the daily tasks for project implementation. The methodology team and the strategic advisory group consist of experts related to research and workforce matters, respectively.

The project manager led the establishment of a workforce reference group, which consisted of 12 Aboriginal people from across NSW undertaking a diploma of nursing scholarship program through the Poche Centre for Indigenous Health and are also working in the health, ageing, or disability sectors. Reflecting the Indigenous research methodology, the purpose of this workforce reference group is to specifically contribute to the co-design and development of the project’s survey and interview questions.

### Engagement and Recruitment

Two groups of participants in NSW will be recruited for this study.

#### Group 1

Group 1 consists of frontline Aboriginal health, ageing, and disability service workers (here on referred to as Aboriginal workers) currently in roles that engage in direct face-to-face client interaction in service delivery in NSW, defined in this study as *frontline service delivery*. The research team aims to recruit a target of 150 frontline workers to complete the survey. This study will exclude those workers who graduated from a registered university to focus on those with vocational qualifications, including diploma certificates or other nonuniversity qualifications. University graduates were excluded because a considerable body of evidence exists regarding workplace experience factors among this group. Furthermore, a large proportion of the ageing and disability workforce consists of people who do not have university qualifications [32].

#### Group 2

Group 2 consists of organizational leaders who employ frontline Aboriginal workers. Aboriginal and non-Aboriginal employers of Aboriginal workers (here on referred to as employers of Aboriginal workers), located anywhere in NSW, will be recruited via the networks of the researchers and publicly available information about their organizations. Staff that may be approached with an invitation to this study include chief executive officers and senior staff of health, ageing, disability, community, and personal service organizations. The research team aims to obtain a target of 50 participants to complete the survey.

The surveys will be sent through the Aboriginal community-controlled organizations, nongovernment industry groups, local health districts, community interagency committees, and nongovernment and for-profit agencies. The alumni of the Poche Centre for Indigenous Health at the University of Sydney and the center’s agency networks will be key recruitment targets. In addition, members of the research team aim to attend Aboriginal community events and major conferences to connect with Aboriginal workers and community organizations where COVID restrictions permit.

In-depth interviews or yarns will be conducted with a subset of 20 Aboriginal workers and 20 employers of Aboriginal workers who have completed the survey and consented to be interviewed. These may be conducted via Zoom or phone, depending on the COVID-19 restrictions.

Considerable effort and numerous engagement activities will be required to achieve the desired targets. Targeted and snowball recruitment will be adopted for both surveys. The strategic advisory group and the project steering committee will also support the research team in recruiting participants.

### Study Design

Surveys will include both closed and open-ended questions and will be conducted on the internet. The survey and interview questions and format will be co-designed with Aboriginal workers and employers of Aboriginal workers to ensure that the language, topics, and issues reflect the lived experience of the workforce and are culturally respectful for Aboriginal workers. Workshops of Aboriginal workers and employers of Aboriginal workers will be held in metro and nonmetro regions to capture the issues and questions for the survey and interviews.

The methodology team, which consists of relevant research team members and the project staff, will advise on the data collection and analysis software and develop procedures that will support the delivery of both the surveys and interviews. The final web-based draft of the questions and procedures will be sent back to the workshop participants for feedback.

### Data Collection

#### Surveys

The first survey will invite Aboriginal workers to share information about their demographics, qualifications, current work role, length of experience, time in their current organization, career path, intention to leave their position, reasons for remaining in their current position, reasons for considering leaving their current position, their career aspirations, and features of an ideal job.

To obtain organizational-level perspectives on the issues related to retention and departure, the second survey will invite employers of Aboriginal workers to share information about their demographics, nature of their organizations, services provided by Aboriginal people, perceptions of turnover of staff in these positions, and factors they perceive promote retention or departure from roles and strategies they have used successfully to promote retention. Rather than aiming for a specific number of agencies, this study aims to ensure a state-wide coverage of the agencies and service types.

A matrix of both participant groups will be developed over the course of the study to ensure that the diversity of sex, age, location, and service type of the participants and agencies are captured.

#### Interviews and Yarning

At the completion of the surveys, participants will be asked if they would be willing to provide further information through participating in a one-to-one telephone interview, Zoom conversation, or in-person yarning session with other local participants if COVID-19 travel and physical distancing restrictions permit. Following Indigenous research methodology, the participants can choose the person on the research team with whom they would like to do the session. Yarning, or yarn ups, is an Indigenous methodology that ensures culturally safe environments for discussing issues that are sensitive and important for Aboriginal people [33]. This method will also be applied to interviews with Aboriginal participants. The direction and contents of the discussions are influenced by the participants. Typically, open-ended questions and *trigger questions* are used to guide discussions, followed by a process of *member checking* as a validation technique [34].

Aboriginal workers will be interviewed separately from employers of Aboriginal workers to ensure that the power relations between these two groups do not inhibit an open discussion. The interviewer will arrange to conduct the interview with the Aboriginal worker at a time requested by the participant to ensure the workers’ confidentiality in the workplace. Interviews will be discontinued once saturation is reached for both groups or the target of 20 participants has been reached. Interviews and yarns will be audio-recorded and transcribed with participants’ consent. Consent via an audio-recording device will be obtained either orally via telephone or web-based platforms or using the written consent form for face-to-face interviews. A participant information script and form will be used by the researcher to inform the participant about the project.

If the participant does not consent to audio recording, 2 team members will be present during the interview to ensure the fidelity of the handwritten record. One will ask the questions or prompts and take notes when feasible, whereas the other will undertake only note-taking. The handwritten notes will then be provided to the participant for checking before inclusion in the research data set. This qualitative component to the research will provide a rich and nuanced understanding of the retention factors identified in the survey and will promote accurate and culturally sensitive interpretation of the results.

### Data Analysis

Descriptive and correlation statistical analyses will be used to examine quantitative data collected via the surveys. Open-ended questions about reasons for staying or leaving their position will be analyzed using the content analysis software NVivo (QSR International), to identify the scope of reasons reported. Similarly, information about career aspirations and ideal jobs will be subjected to thematic analysis. Employer surveys will be analyzed using the same methods.

Interviews and yarns will be analyzed by Aboriginal and non-Aboriginal team members to ensure that cultural nuances are captured using thematic analysis. All transcripts will be managed using the NVivo software. The transcripts will be segmented into content units that will be coded. The codes will be compared within and across the transcripts. The codes will then be clustered together into potential thematic areas. These themes will be named and described based on a cluster of codes. The researchers will verify preliminary findings with the participants to minimize any possible misinterpretations and perform a member check. The analysis approach and the preliminary findings will be reported to the strategic advisory group and the project steering committee for interpretation and feedback and to design approaches for the knowledge to action (KTA) plan. Providing feedback for research activities and preliminary findings is a key part of Indigenous research methodologies.

The survey data will undergo descriptive statistical analysis to describe the frequency of identified barriers to retention and reasons for staying to better understand the motivators for the non–university-qualified Indigenous workforce in relation to the push and pull factors. The frequency of identified barriers to attention and reasons for staying will be correlated with the participants’ intention to leave their current job to explore relationships in the data.

The analysis of the quantitative survey and interview responses will be verified through a second analysis of 20% of the responses by an Aboriginal team member as part of the rigor of the analysis process to minimize potential bias. The thematic analysis of the interviews and yarns will be verified at the code and theme levels. Following this, 20% of the transcripts will be independently coded by a second researcher using the first coder’s code book containing a description of each code. Any discrepancies will be noted and resolved via a consensus between the two coders. The themes will be verified by all the research teams on the project. All research teams will familiarize themselves with the codes and clusters and will agree with the content and names of the themes through a consensus process. This is critical to ensuring that the Aboriginal researchers on the team and the Aboriginal people involved in the governance model of the project bring their interpretation and cultural knowledge to the analysis.

Following these analyses, the survey and interview or yarning results will be analyzed using a triangulation model. As there is very little published literature in this area, it will not be possible to triangulate the results with the existing literature. To ensure transparency of the research findings during the data analysis, the preliminary research findings will be discussed with experts in the field, including the committees used for this project.

### Communication of Results

Aboriginal communities want research to inform the action in their communities. In this study, we define *community* as the *community of practice,* that is, the target workforce and the connected organizations. Through the implementation of this study, we will develop further understandings about the *community of practice* and this in turn will influence the nature of our communication of results. An effective knowledge translation plan can lead to significant changes in service delivery and health outcomes [35]. The following KTA plan details how this study will engage key stakeholders in the research process and the plan for disseminating findings in an ongoing way over the course of the project. The strategic advisory group and the project operations committee will suggest KTA activities that will occur during each year of the project to facilitate community-wide engagement and discussion. On the basis of previous experiences of research conducted by team members in Central Australia, Queensland, and Central Western NSW, the types of KTA activities will be determined in collaboration with the advisory group, project partners, workforce reference group, and project participants. The diversity of stakeholders will ensure that the research findings inform practice and policy.

As shown in Table 2, the team adapted some KTA strategies from research in Central Australia [36]:

1. Community visits: They provide verbal feedback on project activities during their regular community visits.
2. Project newsletters: A quarterly web-based and hardcopy project newsletter written in plain English for distribution to all stakeholders and available on the internet.
3. Presentation at the institutional partner annual general meetings: The annual general meetings are held at culturally and physically accessible venues. An update on the project activities will be provided, and the newsletter will be distributed.
4. Mass media communications: The research team will engage with mass media over the life of the project. For example, to inform the general Aboriginal and Torres Strait Islander communities, the *National Indigenous Times* and local radio stations will be approached to run stories on the project.
5. Policy papers: The team is involved in a range of government committees and working groups in disability affairs. The findings will be reported in policy papers to provide research partners and community organizations with research evidence to inform their respective government advisory roles.

The materials produced for the KTA will be supported by artwork from an Aboriginal artist from NSW and photos gathered during data collection.

**Table 2:** Knowledge translation strategies by stakeholders.

Stakeholder types	Community visits, including verbal feedback	Agency AGM^a^ presentation	Project newsletter	Mass media	Agency policy papers	Final reports
Research participants	Yes^b^	Yes	Yes	Yes	No^c^	Yes
Communities	Yes	Yes	Yes	Yes	No	Yes
Service provider agencies	Yes	Yes	Yes	Yes	No	Yes
Policy makers	No	No	Yes	Yes	Yes	Yes
Academic circles	No	No	Yes	Yes	No	Yes

^a^AGM: annual general meeting.

^b^Yes, this approach will be used with this stakeholder.

^c^No, this approach will not be used with this stakeholder.

## Results

This 3-year (2019-2021) Australian Research Council funded project has fully engaged with Aboriginal workers in the health, ageing, and disability service sectors in NSW. Data collection commenced in 2020, despite the COVID-19 pandemic.

## Discussion

An Aboriginal workforce is essential to the delivery of high-quality, culturally safe health and social services and to address structural barriers to service access. Aboriginal people experience higher rates of disability and chronic health conditions than non-Aboriginal people, which heightens the need for responsive support and services.

A key strength of this study is that it is led by Aboriginal scholars and Aboriginal controlled organizations that apply an Indigenous methodological framework throughout the research process.

This study uses a mixed methods design. The survey and interview questions and model were developed in partnership with Aboriginal health, ageing, and disability, service workers rather than relying only on research publications on the workforce, government policies, and human resources strategies. This design places a strong emphasis on generalizable findings together with an inductive approach that explores employers’ and workers’ lived experience of the Aboriginal health workforce in NSW. Excluding workers who have graduated from university places a strong focus on the workforce who have obtained either school or TAFE or registered training organizations qualifications. Data collection was conducted during the COVID-19 pandemic, and the results will include the unique experiences of Aboriginal workers and employers delivering services in an extremely challenging organizational, community, and personal context.

By identifying the factors that influence the retention of the Aboriginal workforce from yarn ups and surveys completed by Aboriginal workers and their employers, this study will provide a cohesive set of strategies for organizations to apply in improving their retention of Aboriginal workers.

Aboriginal community-controlled organizations and generic mainstream organizations are concerned that research often does not deliver results to their communities. By co-designed research questions, methods, and knowledge dissemination strategies, this project will deliver translatable results to the participating communities. The KTA strategies are designed to share the findings of the research in relevant and accessible ways. This model ensures that the knowledge obtained from this study is returned to the Aboriginal and Torres Strait Islander service sectors.

## Abbreviations

KTA: knowledge to action
NSW: New South Wales
TAFE: Technical and Further Education
